# Evasion of interferon-mediated immune response by arteriviruses

**DOI:** 10.3389/fimmu.2022.963923

**Published:** 2022-08-15

**Authors:** Zhijie Jian, Rui Ma, Ling Zhu, Huidan Deng, Fengqin Li, Jun Zhao, Lishuang Deng, Siyuan Lai, Xiangang Sun, Huaqiao Tang, Zhiwen Xu

**Affiliations:** ^1^ College of Veterinary Medicine, Sichuan Agricultural University, Cheng Du, China; ^2^ Key Laboratory of Animal Disease and Human Health of Sichuan Province, Sichuan Agricultural University, Cheng Du, China; ^3^ College of Animal Science, Xichang University, Xichang, China

**Keywords:** arteriviruses, interferon (IFN), viral proteins, innate immunity, immune evasion

## Abstract

IFN is the most potent antiviral cytokine required for the innate and adaptive immune responses, and its expression can help the host defend against viral infection. Arteriviruses have evolved strategies to antagonize the host cell’s innate immune responses, interfering with IFN expression by interfering with RIG, blocking PRR, obstructing IRF-3/7, NF-κB, and degrading STAT1 signaling pathways, thereby assisting viral immune evasion. Arteriviruses infect immune cells and may result in persistence in infected hosts. In this article, we reviewed the strategies used by Arteriviruses to antagonize IFN production and thwart IFN-activated antiviral signaling, mainly including structural and nonstructural proteins of Arteriviruses encoding IFN antagonists directly or indirectly to disrupt innate immunity. This review will certainly provide a better insight into the pathogenesis of the arthritis virus and provide a theoretical basis for developing more efficient vaccines.

## Introduction

The mammalian immune system can effectively detect and fight against viral infections by inducing the production of type I interferon, which forms the first line of defense. The type I interferon response consists of two parts. The first part is triggered by viral stimulation when cells produce type I interferon and secrete IFN. In the second part of the response, both the IFN-producing cell and adjacent cells sense IFN, leading to the production of IFN-stimulated genes (ISG) (1).

**Figure 1:**
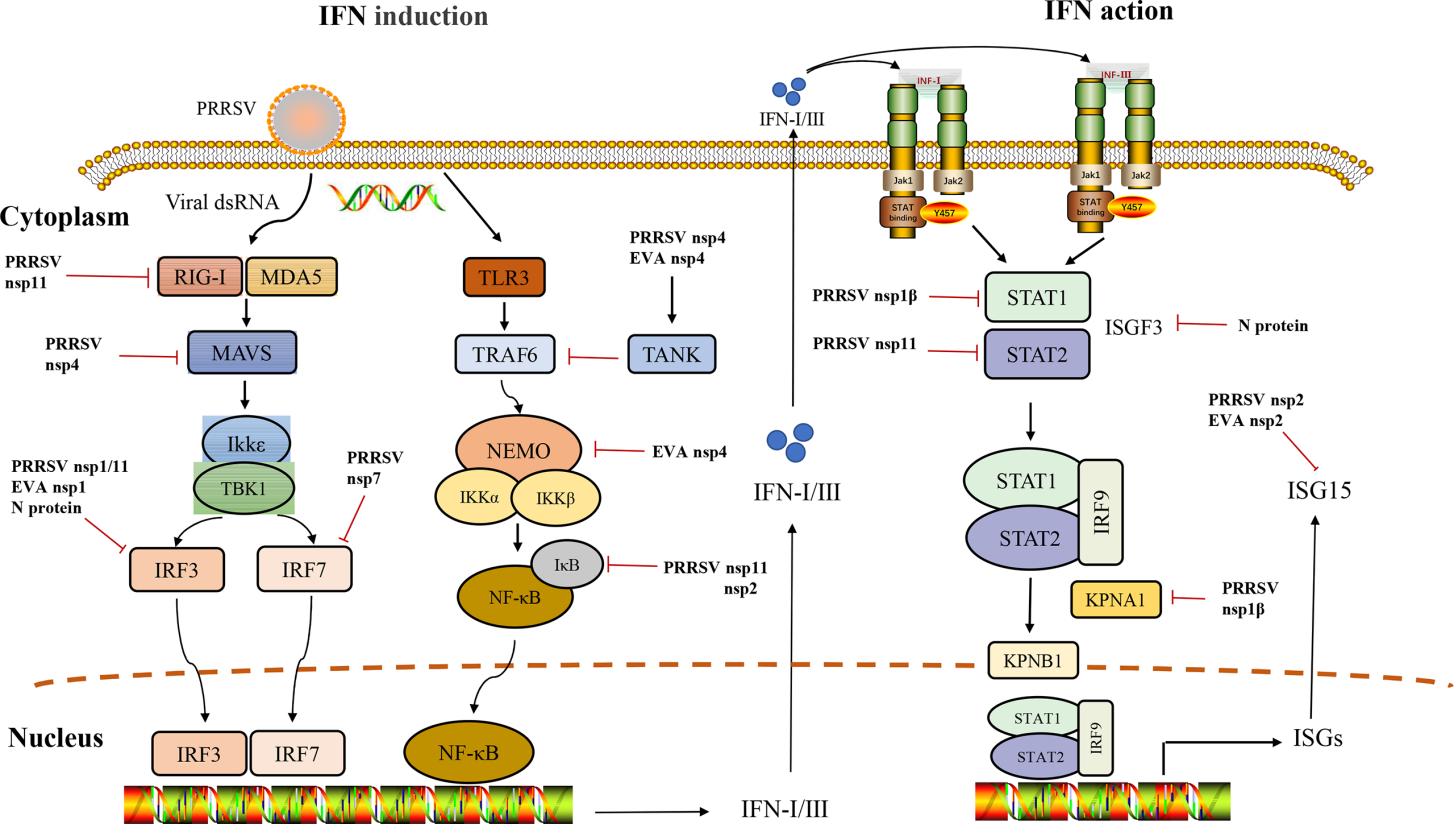
Interference of IFN induction and its downstream signaling pathway by Arteriviruses.

Arteriviruses include porcine reproductive and respiratory syndrome virus (PRRSV), equine arteritis virus (EAV), lactate dehydrogenase-elevating virus (LDV), simian hemorrhagic fever virus (SHFV), and swing possum virus (SPV). They can persist in infected animals, PRRSV can persist for six months in pigs, EAV can persist for life in horses, LDV can usually persist in mice without pathological consequences for the host, and SHFV can show different symptoms in macaques and baboons, with macaques showing fatal hemorrhagic fever but baboons showing only persistent asymptomatic infection (2–5). EAV and PRRSV are considered important pathogens in veterinary studies among these arteriviruses. They can cause significant economic losses in the equine and swine industries, share similar molecular characteristics, and cause reproductive disorders in livestock (6). Therefore, effective Arterivirus control and prevention methods are urgently needed. This review summarizes research advances for the different pathways of anti-IFN responses to Arteriviruses (**Figure 1**). We want to provide creative insights to guide the development of innovative strategies to achieve Arteriviruses prevention and control.

## Overview of interferon response

IFN is a soluble factor discovered in 1957 in viral infections and is named for its ability to interfere in viral replication (7). Interferons are classified into types I, II, and III IFNs (IFN-I, II, and III). In mammals, IFN-I is composed of 19 IFN proteins: 14 IFN-α subtypes (IFN-α1 to α14), IFN-ω, IFN-ε, IFN-τ, IFN-κ, and IFN-β and IFN-I signaling is mediated through the IFN-I receptor (IFNAR), which is a common cell surface receptor. IFN-II family is mainly produced by T lymphocytes and natural killer cells (NK cells), which are mediated by IFNGR (a receptor composed of IFNGR1 and IFNGR2). IFN-III comprises 4 subtypes, IFN-λ1, IFN-λ2, IFN-λ3, and IFN-λ4, and it is mediated by IFNLR (a receptor composed of IFNLR1 and IL10R2) (8–12). IFN-III is associated with IFN-I and IL-10, which have antiviral activity (10). IFN-λ is an epithelial cytokine that limits viral replication in epithelial cells and forms an additional protective layer at mucosal sites (13).

The activation of IFN-I response is divided into three phases: ①pattern recognition receptors (PRRs) on the cell membrane or cytoplasm PRRs recognize pathogen-associated molecular patterns (PAMPs); ②IFN triggers JAK-STAT *via* paracrine or autocrine signaling; ③expression of a large number of antiviral ISG genes, which puts the host into an antiviral state (14).

Most PRRs in the innate immune system of vertebrates can be classified into the following five types: Toll-like receptors (TLRs), retinoic acid-inducible gene I (RIG-I)-like receptors (RLRs), nucleotide oligomerization domain (NOD)-like receptors (NLRs), C-type lectin receptors (CLRs), and absent in melanoma-2 (AIM2)-like receptors (ALRs) (15). We will discuss two classes of viral sensing PRRs in this review. These include TLRs and RLRs, which are important for inducing the type I IFN response. TLRs primarily recognize viral RNA or DNA in the endosomes, and RLRs primarily recognize viral RNA in the cytoplasm. They play a key role in the induction of host IFN expression (16–18). Still, another is a set of structurally unrelated viral DNA sensors (Cyclic GMP-AMP synthase) and IFI16 located in the cytoplasm and/or nucleus, and it also plays a critical role in inducing the expression of host IFN (19). Interferon is normally secreted and binds to cell surface receptors in response to viral infection and activates a JAK/STAT-dependent signaling cascade that produces ISG and puts the cell in a state of resistance (20).

The prolonged infection caused by arteriviruses has a greater association with immune evasion, mainly through the suppression of interferon by various pathways to promote viral proliferation and long-term infection. Clarifying the antagonism between arteriviruses and interferon is important to understand the pathogenesis and find relevant targets as a basis for vaccine development. Therefore, this review will summarize the underlying immune evasion mechanisms by arteriviruses.

## Arteriviruses induce mainly low levels of IFN expression

Arteriviruses use different mechanisms to suppress interferon responses to evade the host’s innate immune response. Early studies have shown that PRRSV infection in pigs leads to a weak induction of the natural immune response. Detection of interferon in alveolar lavage fluid reveals that interferon is maintained at very low levels, suggesting that PRRSV can interfere with IFN-I transcription directly at the level of IFN-β gene transcription in the early stages of infection (21–23). Before the challenge, IFN-pretreatment of pigs *in vivo* reduced PRRSV-induced symptoms. However, it appears that IFN therapy could not rescue PRRSV-infected swine from death, but it extended survival time (24). *In vitro* study, the inhibition of IFNs expression by PRRSV was similarly observed in MARC-145 cells infected with PRRSV and PAMs cells (23, 25).

Similarly, IFN-β production in Equine endothelial cells (EECS) was significantly inhibited after EAV infection, in contrast to SeV infection, which stimulated high levels of IFN-I expression, and EAV infection also significantly inhibited SeV-induced IFN-I production (26). All of the above studies suggest that Arteriviruses induce IFN inhibition, and the main mechanisms responsible for this phenomenon are reviewed next.

## Arteriviruses proteins inhibit the IFN response

Different arteritis virus structural and nonstructural proteins exercise different functions in IFN inhibition (**Table 1**).

**Table 1:** Arteriviruses proteins inhibit IFN downstream signaling pathway.

Arteriviruses proteins	The molecular mechanisms	References
M	PRRSV-specific IFN-γ secretion is correlated with N and M proteins, but the exact mechanism is unclear.	(27)
N	N proteins can inhibit interferon-induced elevated STAT2 levels and ISGF3 nuclear translocation, and their altered localization may also affect the inhibitory activity of IFNs.	(28, 29)
nsp1	Inhibition of IRF3 and IκBα phosphorylation, blocking nuclear translocation of STAT1 and each signaling step upstream of NF-κB activation, cleavage of MAVS and NEMO to antagonize interferon production	(30–35)
nsp2	nsp2 is a potential ISG15 production and binding antagonist and can inhibit Ub and ISG15-dependent innate immune responses.	(36, 37)
nsp4	Targeted cleavage of NEMO and NF-κB activator (TANK) to block NF-κB signaling, cleavage of MAVS and blocking RLR signaling, and inhibition of IFN-β promoter activation.	(33, 34)
nsp7	Inhibits IRF7 expression, thereby downregulating IFN and downstream ISG expression, and promotes viral replication	(38)
nsp11	nsp11 can induce STAT2 degradation directly through the ubiquitin-proteasome degradation pathway and inhibit the NF-κB signaling pathway by de-ubiquitination-dependent activity	(39, 40)

ORF2, 2a, 3, 4, 5, 6, and 7 of PRRSV encode GP2, E, GP3, GP4, GP5, M protein, and N protein, respectively (41). Mechanistic studies of PRRSV antagonism to type I interferon have focused on IFN-α/β, two factors that play a major role in the fight against PRRSV infection. At least two structural proteins (M and N proteins) and four nonstructural proteins of PRRSV, nsp1, nsp2, nsp11, and nsp4, have been identified as exhibiting inhibitory effects on IFN-β promoter activation, with nsp1 showing the strongest inhibitory effect and self-cleavage of nsp1 during infection to produce NSP1α and NSP1β. NSP1β can inhibit IRF3 phosphorylation and NF-κB-dependent nuclear translocation (30, 31). Nsp4 is a 3C-like serine protease that antagonizes type I interferon production by cleaving mitochondria antiviral signaling protein (MAVS) and NF-κB essential regulators (NEMO) (32, 33, 42). PRRSV nsp7 inhibits IRF7 expression, downregulates IFN and downstream ISG expression, and promotes viral replication (38). Nsp11 can suppress the activation of IFN-β by cleaving the mRNA of MAVS (also known as IPS-1, Cardif, and VISA) *via* the endoribonuclease domain (43). PRRSV N protein is distributed in both cytoplasm and nucleus, suggesting that altered localization of N protein may affect its IFNs inhibitory activity (28). Some studies have demonstrated that N protein prevents IFN-β induction like that of nsp2 (44). IFN-γ plays an important role in the immune response against PRRSV. The duration of viremia and the degree of morbidity did not correlate well, but ELISA experiments showed that N, M protein, and nsp2 were indeed associated with PRRSV-specific induction of IFN-γ secretion by lymphocytes (27).

Among the EAV nsp, four nonstructural proteins, nsp1, nsp2, nsp4, and nsp11, have been identified as potential interferon antagonists. It was shown that the homolog of PRRSV nsp1α/β, EAV nsp1, has the strongest ability to inhibit type I IFN synthesis (26). EAV nsp2-encoded papain-like proteinase (PLP2) inhibits Ub- and ISG15-dependent innate immune responses (36). Similarly, EAV nsp4 can inhibit virus-induced IFN-β production by targeting NEMO for protein cleavage, and cleavage occurs at four sites, including E166, E171, Q205, and E349, consistent with PRRSV cleavage sites (34).

## Arteriviruses interference with host interferon induction

### Blocking the recognition of TLR-mediated pathways

The RLRs group consisted of RIG-I, melanoma differentiation-associated gene 5 (MDA5), and Laboratory of Genetics and Physiology 2 (LGP2). RIG-I recognizes the triphosphate and diphosphate at the stem end of dsRNA, which is the hallmark of viral RNA of most RNA viruses (45). MDA5 perceives long dsRNA, which is believed to represent the intermediate replication product of many RNA viruses (46). LGP2, a protein structurally related to both RIG-I and MDA5, appears to be a cofactor for viral RNA sensing by a mechanism that is not completely understood and likely involves making viral RNA more accessible to RIG-I or MDA5 (47). RIG-I and MDA5 are important sensors for IFN-I production in the porcine innate immune system (48). RIG-1 and MDA-5 detect specific viral RNA PAMPs, while LGP2 negatively regulates RIG-I signaling and promotes RNA binding to MDA5 (49). RIG-I-like receptor-mediated type I IFN production plays an important role in the host’s defense against viral invasion (50). dsRNA is a specific secondary structure of viral RNA detected by RIG-I/MDA5 and induces IFN-α/β production through cascade activation of the RLR pathway (51). Viral dsRNA can trigger RIG-I, and the CARD domain of RIG-I interacts with the CARD domain of MAVS, and activation of MAVS recruits multiple downstream signaling components to the mitochondria, leading to activation of κ-B kinase inhibitor ϵ (Iκκϵ) and TANK-binding kinase 1 (TBK1), which in turn causes IRF3 phosphorylation. Phosphorylated IRF3 forms a dimer and translocates to the nucleus, activating transcription of the IFN-I gene (52, 53).

PRRSV infection inhibits IFN-β production mainly by interfering with MAVS activation in the RIG-I signaling pathway (54). The porcine reproductive and respiratory syndrome virus (PRRSV) 3C-like protease (3CLSP), by contrast, cleaves MAVS in a proteasome- and caspase-independent manner at Glu268 (E268/G269). Both cleavage products fail to activate the type I IFN response (55). Further studies showed that the highly pathogenic porcine reproductive and respiratory syndrome virus (HP-PRRSV) protein nsp4 cleaves MAVS and blocks RLR signaling, and causes specific downregulation of the MAVS, but nsp4 in the typical PRRSV strain CH-1a has no effect on MAVS, so this may be a strategy evolved by the virulent strain (32). Nsp11 reduces RIG-I mRNA dependent on its endoribonuclease activity. Nsp11 inhibits IRF3 and NF-κB activity when stimulated with dsRNA analogs and TNF-α, respectively, suggesting that this inhibition also depends on RLR (56).

Recent studies have shown that MDA5 senses the EAV genome to induce IFN expression (57).

### Evasion of the IRF3/7 signaling pathway

Interferon regulatory factors (IRFs) are a family of transcription factors with 9 members identified so far. IRF4, 5, and 6 have no substantial role in IFN regulation and are also not described. IRF-1 and IRF-2 mRNA were expressed in multiple cell types, whereas IRF-8 expression was restricted to myeloid and lymphoid cell lines, and its mRNA was significantly upregulated in response to viral infection or IFN stimulation (58, 59). IRF-9 was originally identified as the DNA-binding subunit of ISGF3 and was proven essential for the antiviral response to IFN-α/β and IFN-γ (60, 61). IRF-3 and IRF-7 are closely related in their primary structure, and recent studies have identified an important and distinct role for these two factors in IFN-α/β gene induction in arteritis virus infection.

It has been suggested that IFN-λ expression is more flexible than IFN-α/β expression, which may allow IFN-III to be expressed in response to a wider range of stimuli than IFN-I, and would potentially make IFN-III expression less sensitive to microbial evasion strategies targeting the IRF pathway (62, 63). IRF3 is a target factor for various viruses and can impair natural immune signaling. Most viruses inhibit IRF3 phosphorylation and thus also IRF3 dimerization and translocation. In the absence of IRF-3 activation and IFN-β production, alternative pathways allow IFN-λ to be induced without IRF-3 activation. IRF-3 is a virus targeting factor and can impair innate immune signaling. Most viruses inhibit IRF3 phosphorylation, dimerization, and nuclear translocation. TBK1 and IKKϵ can induce IRF3 and IRF7 phosphorylation and be affected by K63-linked polyubiquitination (64, 65). The ubiquitin chain may serve as a platform for the assembly of the TBK1 signaling complex, so for TBK1, polyubiquitination of the K63 linkage appears to be important for TLR and RLR-induced IFN production (65, 66). Activated TBK1/IKKϵ phosphorylates IRF3 and/or IRF7 at specific serine residues in the cell membrane, which are subsequently transferred to the nucleus to recruit the coactivator CBP/p300 and form a complex to bind the IRF-3 response element of the IFN-β promoter (PRD I and III) (67–69). Interestingly, IRF7 was induced during IFN signaling at low levels in most cells, suggesting that IRF7 can strongly enhance IFN production (70).

Viral proteins target TBK1 to block IFNβ production by preventing TBK1 activation from MAVS or inhibiting IRF3 activation from TBK1. Once activated, MAVS signaling recruits multiple kinases, ubiquitin ligases, and adapters, leading to phosphorylation and activation of potential transcription factors involved in IFN promoter activation. These transcription factors, IRF factors, especially IRF3 and IRF7, are essential for IFN induction (71, 72). In addition, IRFs are also required for IFN induction during TLR activation. Therefore, it is unsurprising that virally encoded IFN antagonists can inhibit IRFs.

PRRSV nsp1 is the most potent interferon repressor protein among the nonstructural proteins. Studies have shown that the inhibition of type I IFN is due to PRRSV nsp1α/β blocking dsRNA-induced activation of IRF-3. In the presence of nsp1α/β, phosphorylation of IRF-3 and its nuclear translocation occurred normally, but the association of IRF3 with cAMP response element-binding protein(CBP) in the nucleus was inhibited, thereby blocking IRF-3 activation (73, 74). Nsp4 was reported to inhibit IRF-3-mediated activation of the IFN-β promoter, an inhibition derived from the hydrolytic activity of the nsp4 3C-like serine protease (75, 76). Recently, it has been shown that N proteins can inhibit poly(I:C)-mediated IRF-3 phosphorylation and nuclear translocation, thereby suppressing the expression of IFN-β (44). Therefore, IRF3 can be a direct viral target to block IFN production and a key target for vaccine development. IRF7 is another important regulator in the interferon signaling pathway. IRF7 can inhibit the early replication of PRRSV. While PRRSV nsp7 significantly down-regulates IRF7 expression, nsp4 and nsp5 do not down-regulate IRF7 expression. Instead, nsp11 upregulates IRF7 expression, which may result from complex virus-protein interactions (38).

Similarly, EVA nsp1α and NSP1β mediated the inactivation of MAVS, leading to inhibition of IRF-3 activity, which is similar to the role of PRRSV nsp1 (77). It was also found that EAV nsp1 blocked every signaling step upstream of IRF-3, suggesting that EAV nsp1 acts downstream of all these tested steps in this signaling pathway and, interestingly, does not have much effect on the nuclear accumulation of IRF-3, presumably having an effect on the IFN-β promoter in the nucleus (26).

### Blocking TLR-mediated recognition pathway and activation of transcription factor NF-κB

Pathogen-associated molecular patterns in viral RNAs are recognized by various pattern recognition receptors, such as TLR3. TLR-3, -7, -8, and -9 are all capable of inducing type I IFN gene expression, and they exercise the function of detecting different forms of nucleic acids. They scan the extracellular and endosomal space to detect RNA and DNA, detect the viral genome from extracellularly lysis viral particles and initiate signaling cascades that lead to the secretion of IFN and other proinflammatory molecules, such as TLR3 recognition of dsRNA, initiating a TRIF-dependent signaling cascade (52, 78).

Suppressors of cytokine signaling (SOCS) are intracellular family proteins involved in the negative regulation of the immune response (79). Lung epithelial cells can induce IFN-β production and are the first to interact with pathogens, and plasmacytoid dendritic cells (PDCs) can rapidly establish a connection with TLR7 and induce IFN-I expression (80, 81). Recent studies have also shown that SOCS1 and SOCS3 strongly inhibit TLR7-mediated IFN-I production (82, 83) and that PRRSV N proteins can significantly activate SOCS1 promoter activity and induce SOCS1 expression at the protein level in Marc-145 cells, ultimately leading to IFN inhibition (84). Interestingly, TLR3-mediated IFN production after infection with Herpes simplex virus 1 (HSV-1) is cell type-dependent, with TLR3 limiting HSV-1 replication in mouse fibroblasts and CNS-resident cells (neurons, astrocytes), whereas no such protective mechanism is produced in mouse macrophages (85).

TLR3 interacts with TRIF by interacting with upstream adaptors. TRIF undergoes conformational changes and recruits the downstream TNF receptor-associated factor (TRAF)6 (86). The kinase receptor-interacting protein-1 (RIP-1) is part of the signaling pathways downstream of TLR3 and RIG-I. It can interact with TRIF to induce NF-κB activation (87). In its inactive state, the transcription factor NFκB is complexed with its inhibitor IκB (88). Upon stimulation, IκB is phosphorylated by the IκB kinase (IKK) complex, which is composed of two catalytic subunits, such as IKKα and IKKβ, and a regulatory subunit, such as NF-κB essential modulator (NEMO) (89). NF-κB regulates more than 100 genes that play key roles in inflammation, the innate immune response, and the initiation of adaptive immunity (90).

PRRSV nsp1 and nsp2 inhibit the NF-κB signaling pathway to antagonize IFN-β production (91, 92). Nsp1α inhibits the phosphorylation of IκBα, resulting in the nuclear localization of p65 being blocked, thereby aborting NF-κB function, which is associated with its C-terminal 14 amino acids (92). The nsp2 ovarian tumor protease (OUT) structural domain has deubiquitination activity, and IκB degradation is a necessary step for NF-κB activation, which can act on the IκB polyubiquitination process to prevent its degradation and ultimately inhibit NF-κB-mediated production of IFNs (91). PRRSV nsp4 cleaves TRAF family member-associated NFκB activator (TANK), which inhibits TRAF6-mediated NFκB activation (93). PRRSV nsp4 can also block NF-κB signaling targeting NEMO at a single locus E349 (33). Interestingly, the cleaved fragment of NEMO (1-349) still activates IFN and NF-κB promoter production, suggesting that nsp4 may fail to completely prevent NEMO-mediated IFN-β activation *via* cleavage at NEMO E349 (34). PRRSV nsp11 has also been reported to inhibit the NF-κB signaling pathway in response to deubiquitination activity (39).

EAV nsp1 inhibits IFN-β activation mainly through the NF-κB-dependent signaling pathway, which blocks each signaling step upstream of NF-κB activation, but nsp1 has little effect on NF-κB nuclear accumulation. It is speculated that EAV nsp1 may affect the IFN-β promoter in the nucleus (26). It has also been shown that EAV Nsp4 can cleave TANK to inhibit NF-κB expression (93).

## Interference with type I IFN-activated JAK/STAT signaling pathway and antiviral ISGs

Interferons are normally produced and secreted upon viral infection, and secreted IFN binds to the IFN receptor and activates Janus kinase 1 (JAK1) and tyrosine kinase 2 (TYK2) which phosphorylate signal transducers and activators of transcription proteins (STAT1 and STAT2) (94). Phosphorylated STAT1 and STAT2 form heterodimers that bind to IRF9 to form IFN-stimulated gene factor 3 (ISGF3). ISGF3 translocates to the nucleus and binds to the IFN-stimulated response element (ISRE), triggering the expression of hundreds of ISGs with antiviral functions and putting the cell in an antiviral state (20). Antiviral ISG plays a crucial role in eliminating viral infections (95). Many ISGs are signaling molecules or regulatory proteins in innate and adaptive immunity, and their induction of ISGs can further amplify and develop immune responses (including IFN responses) (96, 97).

PRRSV inhibits the IFN-activated JAK/STAT signal transduction and ISG expression in both MARC-145 and PAM cells (29, 98). Further research found that PRRSV nsp1β could block the nuclear translocation of STAT1 and significantly inhibit the expression of ISGs (35). IFN induces IFN-stimulated gene expression by activating phosphorylation of STAT1 and STAT2, which can form a heterotrimer with IRF9 (ISGF3) and translocate to the nucleus. Severe acute respiratory syndrome (SARS) and PRRSV both interfere with the host innate immune responses. Still, mechanisms that block nuclear translocation of ISGF3 are different, and SARS ORF6 can block nuclear translocation of STAT1 by sequestering KPNA2 alone (99). However, no interaction between nsp1β and any KPNAs was found in PRRSV-infected cells. PRRSV VR2385 can inhibit IFN-α signaling in MARC-145 and PAMs by interfering with ISGF3 nuclear translocation, but PRRSV modified live virus (MLV) infection of PAMs can directly activate IFN signaling, suggesting that there may be different effects of IFN induction between the two PRRSV strains, which may provide reference implications for PRRSV vaccine design (35). PRRSV nsp11 can induce STAT2 degradation directly *via* the ubiquitin-proteasome degradation pathway, in which amino acid residue K59 in nsp11 plays a key role but does not depend on endoribonuclease activity (40). Similarly, N proteins can inhibit interferon-induced elevation of STAT2 levels and ISGF3 nuclear translocation (29). PAM cells are affected by IFN-γ and microbial products such as lipopolysaccharide (LPS) and viral infection, and LPS-activated PAMs inhibit PRRSV replication, and genes in the JAK/STAT signaling pathway were found to be significantly upregulated, suggesting that it might play a key role in cellular activation (100).

Among the antiviral ISGs, the best-studied ones are ISG15, 2 ‘,5’-oligoadenylate synthetases (OASs), ribonuclease L (RNaseL), the dsRNA-activated protein kinase (PKR), p56 [ISG56, interferon-induced protein with tetratricopeptide repeats 1 (IFIT1)], and Mx1 (Myxovirus (influenza virus) resistance 1), and IFNs induce upregulation of transcriptional expression of several hundred interferon-stimulated genes (101, 102). ISG15 is a ubiquitin-like antiviral protein [59, 60]. ISG15 conjugation (ISGylation) to substrate proteins follows a process similar to ubiquitin conjugation (103, 104). Many viruses target STAT1 and STAT2 to inhibit the induction of ISG. ISG can inhibit nucleic acid nuclear input and RNA and protein synthesis or enhance viral degradation (102). ISG15 and ISGylation act in different cellular pathways, particularly in regulating antiviral innate immune responses. PRRSV nsp2 was previously identified as a potential antagonist of ISG15 production and ISGylation, overexpression of ISG15 inhibited PRRSV replication in cell culture, and the antiviral activity of interferon was reduced by inhibition of ISG15 binding (37). Interestingly, the pseudoknot region of the 3’ untranslated region (UTR) of the PRRSV genome can be recognized by RIG-I and TLR3 and strongly induces the expression of ISGs in PAMs, and importantly, similar structures predicted for other arterivirus members, including EAV, LDV, and SHFV, also show strong IFN-inducing activity (105).

The interferon-induced PKR plays an important role in antiviral response. PKR mediates translational control by phosphorylating the protein translation initiation factor eIF2α, inhibiting protein synthesis and viral replication (106). The addition inhibitor of PKR (2-AP) restored PRRSV replication in IFN-γ-treated cells (107). Research shows that PRRSV inhibited PKR activation during its early stage infection of PAMs (108).

## Conclusion

Arteriviruses have evolved much to evade the host’s innate immune system to better survive in the host over the long term. The sustained low level of interferon expression is a fundamental reason for their ability to persist. Current studies have identified at least six viral proteins identified as IFN antagonists of PRRSV, further understanding of the immune regulation of viruses and strategies to evade the host immune system is necessary. The development of antiviral drugs can be facilitated by understanding the relationship between Arteriviruses and IFN antagonism to identify key immune evasion proteins. Also, understanding current antiviral strategies can enhance known antiviral pathways and further facilitate the development of safe and effective vaccine strains.

## Author Contributions

LZ, MR, and ZX conceived the scope of the review article writing, HD and FL assisted with language revisions. JZ, LD, SL, XS, and HT assisted with reviewing relevant literature. ZJ wrote the review with the help of other authors. All authors contributed to the article and approved the submitted version.

## Funding

This article was supported by the Sichuan Province’s “14th Five-Year Plan” Sichuan Pig Major Science and Technology Project (No. 2021ZDZX0010) and the Key R&D Program in Rural Areas of Sichuan Provincial Department of Science and Technology (No. 2020YFN0147).

## Conflict of Interest

The authors declare that the research was conducted in the absence of any commercial or financial relationships that could be construed as a potential conflict of interest.

## Publisher’s note

All claims expressed in this article are solely those of the authors and do not necessarily represent those of their affiliated organizations, or those of the publisher, the editors and the reviewers. Any product that may be evaluated in this article, or claim that may be made by its manufacturer, is not guaranteed or endorsed by the publisher.
